# Late-Onset Neutropenia after Rituximab Treatment for Adult-Onset Nephrotic Syndrome

**DOI:** 10.1155/2019/3580941

**Published:** 2019-03-05

**Authors:** Mayuko Yamazaki, Hidekazu Sugiura, Yosuke Iwatani, Mizuki Kyoda, Hirohiko Nokiba, Nobuyuki Amemiya, Kosaku Nitta, Ken Tsuchiya

**Affiliations:** ^1^Department of Nephrology, Division of Medicine, Saiseikai Kurihashi Hospital, Saitama, Japan; ^2^Department of Nephrology, Tokyo Women's Medical University, Tokyo, Japan; ^3^Department of Blood Purification, Kidney Center, Tokyo Women's Medical University Tokyo, Japan

## Abstract

A 41-year-old woman developed nephrotic syndrome at the age of 32 and was diagnosed with minimal change nephrotic syndrome based on a renal biopsy. Although remission was achieved with administration of prednisolone (PSL) and cyclosporine, the nephrotic syndrome recurred. She was also started on rituximab (RTX). She developed late-onset neutropenia after RTX treatment (R-LON) and improved 17 days later. Although the majority of R-LON cases undergo spontaneous remission, cases of death have been reported. This report is intended to warn about R-LON, since the use of RTX for adult-onset nephrotic syndrome is expected to increase in the future.

## 1. Introduction

Rituximab (RTX) binds to CD20 which is expressed on the surface of human B cells and produces complement-dependent cytotoxic and antibody-dependent cell-mediated cytotoxic effects [1]. RTX is used for B-cell non-Hodgkin's lymphoma but has been recently reported as an effective treatment for childhood-onset refractory nephrotic syndrome, adult-onset nephrotic syndrome [2–4], antibody-mediated rejection of ABO-incompatible kidney transplant, rheumatoid arthritis, and autoimmune diseases [5–10].

This report describes a case of grade 3 neutropenia that occurred after 188 days of RTX treatment for adult-onset nephrotic syndrome.

## 2. Case Report

Patient is a woman whose age is 41 years.

History of present illness is as follows: lower leg edema appeared 1.5 months after the delivery of her second child. The patient was hospitalized on the middle of October 2007 with a diagnosis of nephrotic syndrome. Severe edema and pleural effusion were observed, and the patient was started on hemodialysis and 500 mg/day of methylprednisolone for 3 days. After 3 days of administration, 60 mg/day of prednisolone (PSL) was initiated. Type I partial remission was achieved 24 days later, and the patient was taken off dialysis and discharged on the early November. After discharge, while the patient was being tapered off PSL, nephrotic syndrome recurred in June of 2008 when the PSL dosage was reduced to 17.5 mg/day. The PSL dosage was increased to 50 mg/day with the addition of 50 mg/day of cyclosporine, and the patient promptly tested negative for proteinuria. The PSL dose was gradually reduced again; however, the patient experienced a second recurrence of nephrotic syndrome when the dosage was decreased to 7.5 mg/day in August 2011. The patient underwent a renal biopsy and was diagnosed with minimal change nephrotic syndrome. Serology results (normal values for C3 at 102 mg/dL and C4 at 37 mg/dL and negative tests for antinuclear antibody and anti-dsDNA antibody) as well as negative immunostaining of the renal biopsy specimen (including for IgG, IgA, IgM, C3, C4, and C1q) excluded a diagnosis of lupus nephritis or other nephritis. The PSL dose was again increased to 50 mg/day, and complete remission was rapidly achieved. The patient was being tapered off PSL when recurrent nephrotic syndrome occurred for the third time. Thus, the patient was deemed to have recurrent steroid-dependent nephrotic syndrome, in which PSL dose reduction is difficult.

**Figure 1:**
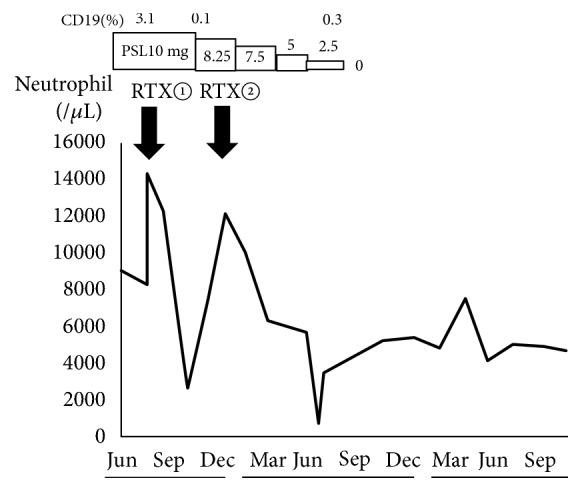
Clinical course. PSL: prednisolone. RTX: rituximab.

In July of 2016, 500 mg of RTX was administered, after which a complete remission of nephrotic syndrome was maintained even with a reduced dose of PSL. Although mild neutropenia was noted (2,579/*μ*L neutrophil count) on day 79 of RTX treatment, the neutrophil count rapidly increased to 12284/*μ*l when steroids were administered (Figure 1). The second 500 mg dose of RTX was administered in December of 2016. In July of 2017, a further decrease in the neutrophil count of 619/*μ*L was observed, which led to the patient's hospitalization.

Physical findings upon admission were height 155 cm, body weight 46.2 kg, blood pressure 90/58 mmHg, pulse 82/min, body temperature 36.3°C, no palpebral conjunctival anemia, no yellowing of the bulbar conjunctiva, clear lung sounds, no heart murmur, flat and soft abdomen, no abdominal tenderness, and no edema of the limbs.

Test findings upon admission (Table 1) were grade 3 neutropenia (neutrophil count < 500–1000/*μ*L). CRP was negative at 0.05 mg/dL.

Course after hospitalization was as follows: despite the presence of neutropenia, no clear signs of infection were seen; thus, granulocyte-colony stimulating factor (G-CSF) treatment was not performed. After admission, the neutrophil count gradually increased and improved to 3,471/*μ*L in the late July (Figure 1). The patient's nephrotic syndrome has remained in complete remission without medication for more than 18 months after the second RTX treatment. Therefore, there has not been an indication for further RTX.

## 3. Discussion

Commonly known adverse events of RTX include infusion reaction, infection, lymphocytopenia, progressive multifocal leukoencephalopathy, fulminant hepatitis caused by the hepatitis B virus, and exacerbation of hepatitis. Recent reports on R-LON occurring more than 4 weeks after the last dose of RTX exist. Although no widely accepted standard definition of R-LON has been established, the condition is defined as “otherwise unexplained grade ≥3 neutropenia (<1,000/*μ*L) according to National Cancer Institute Common Toxicity Criteria occurring 3–4 weeks after the final RTX dose” [11].

R-LON is frequently reported in patients with B-cell lymphoma but has also been reported in cases of rheumatoid arthritis, systemic lupus erythematosus [9], overlap syndrome [8], renal transplantation [5, 7] and antineutrophil cytoplasmic antibody- (ANCA-) associated vasculitis [6]. The incidence is 3%-27% in B-cell lymphoma [11], 1.3%-2.3% in rheumatoid arthritis and autoimmune diseases [9], and 42%-70% in autologous stem cell transplantation [7]. The wide range of incidences is attributable to the diversity in patient backgrounds and treatments, inconsistent definitions of R-LON in the literature, and differences in the frequency of post-RTX administration blood sampling.

Although reports of R-LON in childhood-onset nephrotic syndrome exist [12], this is the first report of R-LON in a case of adult-onset nephrotic syndrome.

The median time interval between the final dose of RTX and R-LON onset is 38-175 days [11]. In the present case, neutropenia was observed 188 days after the final RTX dose. Furthermore, the neutrophil count 30 days after the first RTX dose was 12,284/*μ*L but decreased to 2,579/*μ*L 79 days after the first RTX dose. It is possible that more severe levels of neutropenia occurred but were undetected, as the patient was followed up at the outpatient clinic only every 7 weeks.

No consensus has been reached regarding the risks of R-LON onset. Arai et al. reported that stage ≥3 progressive B-cell lymphoma at the time of initiating treatment and age ≥60 years are risk factors [13]. Moreover, recent studies have reported that individuals with a specific IgG Fc receptor polymorphism (FC*γ*RIIIa 158V/F) are at a higher risk for developing R-LON [13–16].

Although the precise mechanism underlying R-LON onset is yet to be established, the proposed hypothesis is one of two broadly classified options: (1) an immunological process or (2) a hematopoietic disorder [15]. Some B-cell clones can reportedly produce autoantibodies to neutrophils and myeloid progenitors during the recovery phase following an RTX-induced decrease of normal B-cell count [17, 18]. Other reports have described that the increase of stromal-derived factor-1, which plays an important role in B-cell count recovery, disturbs the migration of granulocytes from the bone marrow to the peripheral blood, among other actions [5, 13, 17]. The patient in the present case, however, tested negative for antineutrophil antibodies.

In the present case, the neutrophil count improved spontaneously. Although R-LON is generally asymptomatic and is known to spontaneously go into remission, reported cases of complicating infection that requires G-CSF or antibiotic treatment also exist [5, 6, 9–11, 13]. Deaths have also occurred due to severe infection [7, 19]. Caution is thus required to prevent R-LON onset. Although no consensus has been reached regarding G-CSF administration, G-CSF is commonly administered in grade 4 R-LON, regardless of infection. In many cases, myeloid progenitors with G-CSF receptors are maintained. Thus, prompt recovery of neutrophil count can be expected by administering G-CSF [18].

In cases of RTX readministration after the onset of R-LON [6, 9–11, 13, 19], reports of both R-LON recurrence and nonrecurrence exist. Since it is rare for cases of R-LON recurrence to become severe, RTX readministration is still considered possible. However, increasing the cumulative dose of RTX has also been reported to increase the risk of R-LON onset [20]. Many aspects of R-LON remain unknown, making it difficult to predict the risk or moment of onset. Thus, careful observation is required following RTX readministration.

## 4. Conclusions

We encountered a case of R-LON induced by administration of RTX for adult-onset nephrotic syndrome. Although spontaneous remission is frequently observed in R-LON, severe infection can also ensue. Patients should therefore be followed up carefully for signs of R-LON onset when RTX is administered for adult-onset nephrotic syndrome.

## Figures and Tables

**Table 1:** Laboratory findings on admission.

*Urinalysis *		*Blood chemistry*	
Protein	(-)	TP	6.2 g/dL
Blood	(-)	Alb	4.0 g/dL
Glucose	(-)	Na	141 mEq/L
Keton	(-)	K	4.4 mEq/L
*Blood cell count*		Cl	108 mEq/L
		BUN	9 mg/dL
WBC	1770 /*μ*L	Cr	0.66 mg/dL
Neut	35.0 %	AST	16 U/L
Lymp	11.7 %	ALT	15 U/L
Mono	8.6 %	LDH	151 U/L
Eosino	0.5 %	*γ*GTP	17 U/L
Baso	0.5 %	CRP	0.05 mg/dL
Hb	14.1 g/dL	*Others*	
Ht	41.2 %	*β*-D glucan	<2.161 pg/mL
Plt	195×10^3^ /*μ*L	CD19	0.4 %
		Anti-Neutrophil antibody	(-)

## References

[1] Takei T., Iwabuchi Y., Nitta K. (2014). Rituximab on steroid-dependent and steroid –resistant nephrotic syndrome in adult. *Jin To Touseki (Kidney and Dialysis)*.

[2] Sugiura H., Takei T., Itabashi M. (2011). Effect of single-dose rituximab on primary glomerular diseases. *Nephron Clinical Practice*.

[3] Iwabuchi Y., Takei T., Moriyama T., Itabashi M., Nitta K. (2014). Long-term prognosis of adult patients with steroid-dependent minimal change nephrotic syndrome following rituximab treatment. *Medicine (Baltimore)*.

[4] Miyabe Y., Takei T., Iwabuchi Y., Moriyama T., Nitta K. (2016). Amelioration of the adverse effects of prednisolone by rituximab treatment in adults with steroid-dependent minimal-change nephrotic syndrome. *Clinical and Experimental Nephrology*.

[5] Kabei K., Uchida J., Iwai T. (2014). Late-onset neutropenia and acute rejection in ABO-incompatible kidney transplant recipients receiving rituximab and mycophenolate mofetil. *Transplant Immunology*.

[6] Knight A., Sundström Y., Börjesson O., Bruchfeld A., Malmström V., Gunnarsson I. (2016). Late-onset neutropenia after rituximab in ANCA-associated vasculitis. *Scandinavian Journal of Rheumatology*.

[7] Ahmadi F., Dashti-Khavidaki S., Khatami M.-R., Lessan-Pezeshki M., Khalili H., Khosravi M. (2017). Rituximab-related late-onset neutropenia in kidney transplant recipients treated for antibody-mediated acute rejection. *Experimental and Clinical Transplantation*.

[8] Akram Q., Roberts M., Oddis C., Herrick A., Chinoy H. (2016). Oddis, Arianne Herrick, Hector Chinoy. Rituximab-induced neutropenia in a patient with inflammatory myopathy and systemic sclerisus overlap disease. *Reumatologia*.

[9] Salmon J. H., Cacoub P., Combe B. (2015). Late-onset neutropenia after treatment with rituximab for rheumatoid arthritis and other autoimmune diseases: data from the autoimmunity and rituximab registry. *RMD Open*.

[10] Reitblat O., Wechsler A., Reitvlat O. (2015). Rituximab-related late-onset neutropenia in patients with rheumatic diseases: successful re-challenge of the treatment. *American Journal of Case Reports*.

[11] Wolach O., Bairey O., Lahav M. (2010). Late-onset neutropenia after rituximab treatment: case series and comprehensive review of the literature. *Medicine*.

[12] Hiramoto R., Matsumoto S., Eguchi H. (2009). A case of a 7-year-old boy with steroid-dependent nephrotic syndrome who developed severe neutropenia three months after the administration of rituximab. *Nihon Shoni Jinzobyo Gakkai Zasshi (Japanese Journal of Pediatric Nephrology)*.

[13] Arai Y., Yamashita K., Mizugishi K. (2015). Risk factors for late-onset neutropenia after rituximab treatment of B-cell lymphoma. *International Journal of Hematology*.

[14] Moore D. C. (2016). Drug-induced neutropenia: A focus on rituximab-induced late-onset neutropenia. *P&T*.

[15] Tesfa D., Palmblad J. (2011). Late-onset neutropenia following rituximab therapy: Incidence, clinical features and possible mechanisms. *Expert Review of Hematology*.

[16] Keane C., Nourse J. P., Crooks P. (2012). Homozygous FCGR3A-158V alleles predispose to late onset neutropenia after CHOP-R for diffuse large B-cell lymphoma. *Internal Medicine Journal*.

[17] Chaiwatanatorn K., Lee N., Grigg A., Filshie R., Firkin F. (2003). Delayed-onset neutropenia associated with rituximab therapy. *British Journal of Haematology*.

[18] Arai Y., Yamashita K. (2014). Characteristics and clinical significance of late-onset neutropenia after rituximab treatment. *Ketsueki Naika*.

[19] Aguiar-Bujanda D., Blanco-Sánchez M. J., Hernández-Sosa M. (2015). Late-Onset Neutropenia after Rituximab-Containing Therapy for Non-Hodgkin Lymphoma. *Clinical Lymphoma, Myeloma & Leukemia*.

[20] Cattaneo C., Spedini P., Casari S. (2006). Delayed-onset peripheral blood cytopenia after rituximab: Frequency and risk factor assessment in a consecutive series of 77 treatments. *Leuk Lymphoma*.

